# Flow cytometric analysis of lymphocyte profiles in mediastinal lymphadenopathy of sarcoidosis

**DOI:** 10.1371/journal.pone.0206972

**Published:** 2018-11-19

**Authors:** Ken Akao, Tomoyuki Minezawa, Naoki Yamamoto, Takuya Okamura, Takahiro Inoue, Kumiko Yamatsuta, Sakurako Uozu, Yasuhiro Goto, Masamichi Hayashi, Sumito Isogai, Masashi Kondo, Kazuyoshi Imaizumi

**Affiliations:** 1 Department of Respiratory Medicine, Fujita Health University School of Medicine, Toyoake, Aichi, Japan; 2 Regenerative Medicine Support Promotion Facility, Center for Research Promotion and Support, Fujita Health University, Toyoake, Aichi, Japan; Oklahoma Medical Research Foundation, UNITED STATES

## Abstract

Lymphocyte profiles in mediastinal lymph nodes may reflect the immune status of patients with sarcoidosis. Endobronchial ultrasound-guided transbronchial needle aspiration (EBUS-TBNA) is useful for the diagnosis of diseases with mediastinal lymphadenopathy including sarcoidosis. The purpose of this study was to determine lymphocyte profiles of lymph nodes in sarcoidosis by analyzing EBUS-TBNA samples. We prepared single cell suspensions from EBUS-TBNA samples of mediastinal lymph nodes from patients with sarcoidosis or lung cancer and analyzed surface markers (CD3, CD4, CD8, CD19, CD25) and FoxP3 expression in the resultant lymphocytes using flow cytometry. We studied 26 patients with sarcoidosis and 16 with lung cancer with mediastinal lymph node metastases. In sarcoidosis, the CD4/CD8 ratio was significantly more elevated in lymph nodes than in bronchoalveolar lavage fluid (P<0.001), although both were strongly correlated. The CD4/CD8 ratio was significantly higher in stage I than in stage II both in the BAL fluid and lymph nodes. When compared with lung cancer lymph node metastasis, the CD4/CD8 ratio was significantly higher in sarcoidosis, whereas the CD3/CD19 ratio was significantly higher in lung cancer. The proportion of regulatory T cells (CD4^+^, CD25^+^, FoxP3 ^high^) did not differ between sarcoidosis and lung cancer samples. Lymphocyte profiles in mediastinal lymphadenopathy can be analyzed by flow cytometry of EBUS-TBNA samples. These findings might help elucidate the immunopathology of sarcoidosis.

## Introduction

Although CD4-positive T-lymphocytes are thought to play an important role in sarcoidosis, its pathogenesis has not been fully elucidated, although the excessive activation of CD4 positive T lymphocytes is a hallmark of sarcoidosis [1]. An elevated ratio of CD4/CD8 positive T-lymphocytes in bronchoalveolar lavage (BAL) fluid is suggestive for sarcoidosis diagnosis [2]. However, little is known about lymphocyte dynamics in the affected lymph nodes in sarcoidosis. Although previous reports reported the lymphocyte profile in sarcoidosis lymph nodes including the CD4/CD8 ratio, the results were inconsistent among these reports [3,4]. With recent advances in respiratory endoscopic technologies including endobronchial ultrasound-guided transbronchial needle aspiration (EBUS-TBNA), the diagnostic accuracy for various mediastinal or hilar lymphadenopathies has been markedly improved. EBUS-TBNA samples are available for histological diagnosis and for molecular diagnosis or immunohistochemical analyses [5,6,7]. Recent studies showed gastrointestinal endoscopic ultrasound guided fine needle aspiration (FNA) biopsy is an established procedure for the diagnosis of lymphoma [8,9,10]. FNA-biopsy samples can be used for flow cytometric analysis, which is essential for the diagnosis of lymphoma. The purpose of this study was to analyze lymphocyte profiles in sarcoidosis lymph nodes by analyzing EBUS-TBNA samples using flow cytometry. We investigated the ratio of T-lymphocytes/B-lymphocytes, CD4/CD8 T-lymphocytes and regulatory T cells (Treg)/CD4 T-lymphocytes in affected lymph nodes of sarcoidosis patients. Comparative analyses of lymphocyte profiles in BAL fluid and lymph nodes were also performed. To elucidate the specific lymphocyte profile of sarcoidosis, we also compared the lymphocyte profile between sarcoidosis lymph nodes and lung cancer metastatic lymph nodes.

## Materials and methods

### Subjects

From November 2017 to March 2018, 52 patients underwent EBUS-TBNA for the diagnosis of mediastinal lymphadenopathy in Fujita Health University Hospital. Among these patients, written informed consent could not be received from four patients and one patient refused to participate in the study. Thus, 47 patients were included in this study. Five patients with a final diagnosis that was not sarcoidosis or lung cancer metastases were excluded. Finally, 26 cases of sarcoidosis and 16 cases of lung cancer with mediastinal lymph node metastases were analyzed. The patient characteristics are shown in Table 1.

**Table 1:** Patients background.

	Sarcoidosis	Lung cancer
Patient number	26	16
Gender		
male/female	13/13	11/5
Age		
median (range)	47(20–77)	75(44–83)
Smoking status		
current/ex*/never smoker	12/6/8	5/11/0
Lymph node biopsied [11]		
#4 / #7 / #10 / #11**	7/15/1/3	7/7/1/1

current, current smoker; ex, ex-smoker.

**The number of mediastinal lymph node in this article is based on an international lymph node map in the TNM classification for lung cancer. #2,upper paratracheal; #4,lower tracheal; #7,subcarinal; #10,hilar; #11,interlobar lymph node.

The ethics committee of Fujita Health University approved this study protocol, which was conducted in accordance with the tenets of the Declaration of Helsinki (approval number FH-14-035). The registration number of this study is UMIN 000021850. Written informed consent was obtained from all participants in this study.

### Bronchoscopy

For EBUS-TBNA, we used a convex probe endobronchial ultrasound (Olympus UC260FW-OL8, Olympus Co Ltd, Tokyo, Japan) and dedicated fine needles (NA-201SX-4022, 22 gauge, Olympus, Tokyo, Japan). We selected the target lymph node station according to the chest CT or positron emission tomography (PET)-CT findings. More specifically, among #2 (upper paratracheal), #4 (lower tracheal), #7 (subcarinal), and #10 (hilar) lymph nodes, an enlarged lymph node (short axis > 1 cm) with high uptake of ^18^F fluorodeoxyglucose (FDG) was selected according to the physician’s choice [11]. Under a real-time echoic view, TBNA was performed five to six times for each patient. We did not use rapid onsite evaluation. When we performed bronchoscopy on patients suspected of sarcoidosis, we performed bronchial alveolar lavage (BAL) immediately after EBUS-TBNA. BAL procedures and the interpretation of specimens were carried out according to the standard method of the American Thoracic Society [12]. In brief, after a bronchoscope (Olympus 260 or Olympus 1T-260, Olympus Ltd. Tokyo, Japan) was wedged into the target segment bronchus, 50 ml of saline was administered to the target segment (mostly middle lobe or lingular lobe) via the working channel three times. Instillation and retrieval of saline were performed manually using a 50 ml syringe. Retrieved BAL fluid was filtered and collected.

### Analyses of lymphocyte profile by flow cytometry

Two to three pieces of EBUS-TBNA specimens were collected and used for flow cytometry. TBNA samples were pushed out from the puncture needle into high glucose Dulbecco’s modified Eagle medium (DMEM) containing 5% fetal bovine serum (FBS). Phosphate buffered saline (PBS) was added to the samples, mixed by inversion, and centrifuged. The cell pellet was washed and centrifuged twice. Then the pellet was minced on a petri dish with a scalpel blade. The minced samples were dispersed into a single cell suspension with repeated pipetting. For hemolysis treatment, (EasyLyse Agilent Technologies, Inc., Santa Clara, CA, USA) was added to samples and stirred, followed by incubation at room temperature for 15 minutes according to the manufacturer’s instruction. After washing again with PBS, samples were stirred and filtered. The cell count of each sample was calculated by the Trypan Blue exclusion method. We prepared a single cell suspension sample that contained 1–5×10^7^ cells/ml from each sarcoidosis sample, and 5–10×10^5^ cells/ml from each lung cancer sample. Each dispensed sample was labeled with anti-CD3-PC5 + anti-CD4-FITC + anti-CD8-PE antibody (Beckman Coulter Inc., CA, diluted 1:10), anti-CD3-FITC + anti-CD19-PE antibody (Beckman Coulter Inc., diluted 1:10), or anti-CD4-FITC + anti-CD25-PE + anti-FoxP3-APC antibody (Miltenyi Biotec Inc., Bergisch, Gladbach, Germany, diluted 1:10). For FoxP3 labeling, cell fixation and permeabilization treatments were performed. For the analyses of BAL specimens, a mixture of monoclonal anti-CD3-PC5, anti-CD4-FITC, and anti-CD8-PE antibodies (BD Multitest, BD Biosciences, San Jose, CA) were used for flow cytometry. We checked cell counts and fractionation on each BAL sample. For each analysis using different antibody combinations, 1–3×10^4^ cells were counted. All flow cytometric analyses were performed using FACS Calibur (BD Biosciences).

### Statistical analysis

For the analysis of data from two related samples, the P value was calculated by Wilcoxon signed-rank test. The Mann-Whitney *U*-test was used to compare two independent groups of data. Regarding the correlation between two groups of corresponding data, the P value was calculated by Spearman’s rank correlation coefficient. Linear regression analysis was also used to calculate r^2^. All statistical analyses were performed using JMP (SAS Institute, Tokyo, Japan).

## Results

### Flow cytometric analysis of EBUS-TBNA samples

**Fig 1:**
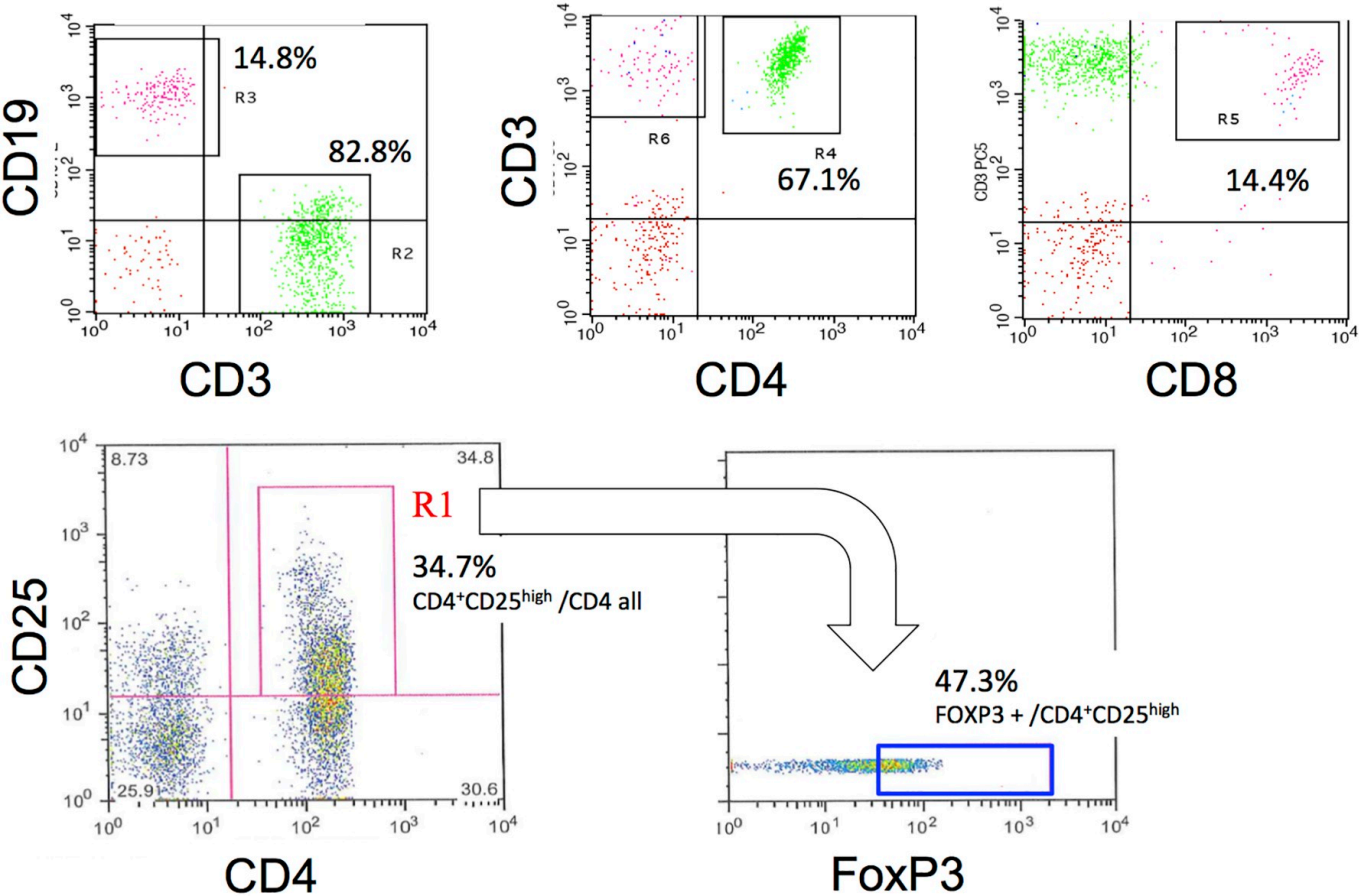
A representative schema of flow cytometric analysis of lymph nodes. A single cell suspension sample from sarcoidosis or lung cancer lymph nodes was labeled with anti-CD3, anti-CD19, anti-CD4, and anti-CD8 antibodies. To detect Treg, CD4 and CD25 positive cells were sorted followed by cell fixation and permeabilization treatment with anti-FoxP3 antibody labeling. CD4+/CD25+ cells with high FoxP3 expression were determined as Treg.

Fig 1 shows the flow cytometry analysis of a TBNA sample from a representative sarcoidosis case. Detailed clinical, radiological, and pathologic findings of sarcoidosis cases are shown in Table 2. Flow cytometry clearly differentiated between CD3+ and CD19+ cells (R2 and R3 respectively, Fig 1). The analysis also clearly identified CD4 T-lymphocytes (R4, Fig 1) and CD8 T-lymphocytes (R5, Fig 1) in this sample. We also identified CD4+/CD25+ lymphocytes and FoxP3 highly positive cells in this population. We determined the CD4/CD25 positive and FOXP3 high lymphocyte population as Treg (Fig 1, S1 Fig). In sarcoidosis, lymphocytes in lymph nodes consisted of 56.6% CD4+ cells (SD: 13.8%), 9.60% CD8+ cells (SD: 5.1%), and 29.5% CD19+ cells (SD 15.0%) on average.

**Table 2:** Sarcoidosis; clinical, radiologic, and pathologic findings.

Case	Age/Sex*	Clinical features§	CRS¶	Past medical history	Steroid**	Biopsy§§
1	63/M	BHL Diffuse micronodules	II	Cerebral hemorrhage Colon cancer	None	TBB EBUS-TBNA
2	40/F	BHL	I	Cervical dysplasia	None	EBUS-TBNA
3	30/M	BHL Diffuse micronodules	II	None	None	TBB EBUS-TBNA
4	37/M	BHL	I	Goiter Appendicitis	None	EBUS-TBNA
5	74/F	BHL Diffuse micronodules Cutaneous nodules	II	Hypertension	None	TBB EBUS-TBNA Skin Biopsy
6	46/M	BHL Uveitis	I	Dyslipidemia, Appendicitis	None	EBUS-TBNA
7	61/F	BHL	I	Stomach cancer	None	EBUS-TBNA
8	20/F	BHL Uveitis	I	None	None	EBUS-TBNA
9	25/M	BHL Diffuse micronodules	II	None	None	TBB EBUS-TBNA
10	67/F	BHL Complete AV block Cutaneous nodules	II	Aortic stenosis Mitral stenosis	None	EBUS-TBNA Skin Biopsy
11	65/F	BHL Diffuse micronodules Uveitis	II	Lower extremity varices	None	EBUS-TBNA
12	34/M	BHL	I	Appendicitis	None	EBUS-TBNA
13	76/F	BHL	I	Diabetes Dyslipidemia	None	EBUS-TBNA
14	69/M	BHL	I	Gall bladder cancer	None	EBUS-TBNA
15	38/M	BHL Multiple GGO	II	Sinusitis	None	EBUS-TBNA
16	45/F	BHL Diffuse micronodules	II	Colon cancer Uterine fibroid	None	TBB EBUS-TBNA
17	65/F	BHL	I	Diabetes Rectal cancer	None	EBUS-TBNA
18	65/F	BHL	I	Endometrial cancer	None	EBUS-TBNA
19	47/F	BHL Diffuse micronodules	II	Diabetes	None	EBUS-TBNA
20	35/M	BHL Diffuse micronodules	II	None	None	TBB EBUS-TBNA
21	56/F	BHL	I	Herpes zoster	None	EBUS-TBNA
22	42/M	BHL	I	None	None	EBUS-TBNA
23	36/M	BHL Diffuse micronodules Uveitis	II	None	None	EBUS-TBNA
24	65/M	BHL Consolidation	II	Autoimmune pancreatitis	DEX 0.5mg/d	EBUS-TBNA Lobectomy
25	77/F	BHL AV block (Mobitz II)	I	Diabetes Dyslipidemia	None	EBUS-TBNA
26	40/M	BHL	I	None	None	EBUS-TBNA

*M, male; F, female

^§^ Clinical features includes radiological findings and extrapulmonary lesions; BHL, bilateral hilar lymphadenopathy; GGO, Ground-glass opacities; AV block, atrioventricular block.

^¶^CRS, chest radiographic stage.

**DEX, dexamethasone

^§§^TBB, transbronchial lung biopsy;

EBUS-TBNA, endobronchial ultrasound-guided transbronchial needle aspiration.

### Lymphocyte profile in sarcoidosis lymphadenopathy

**Fig 2:**
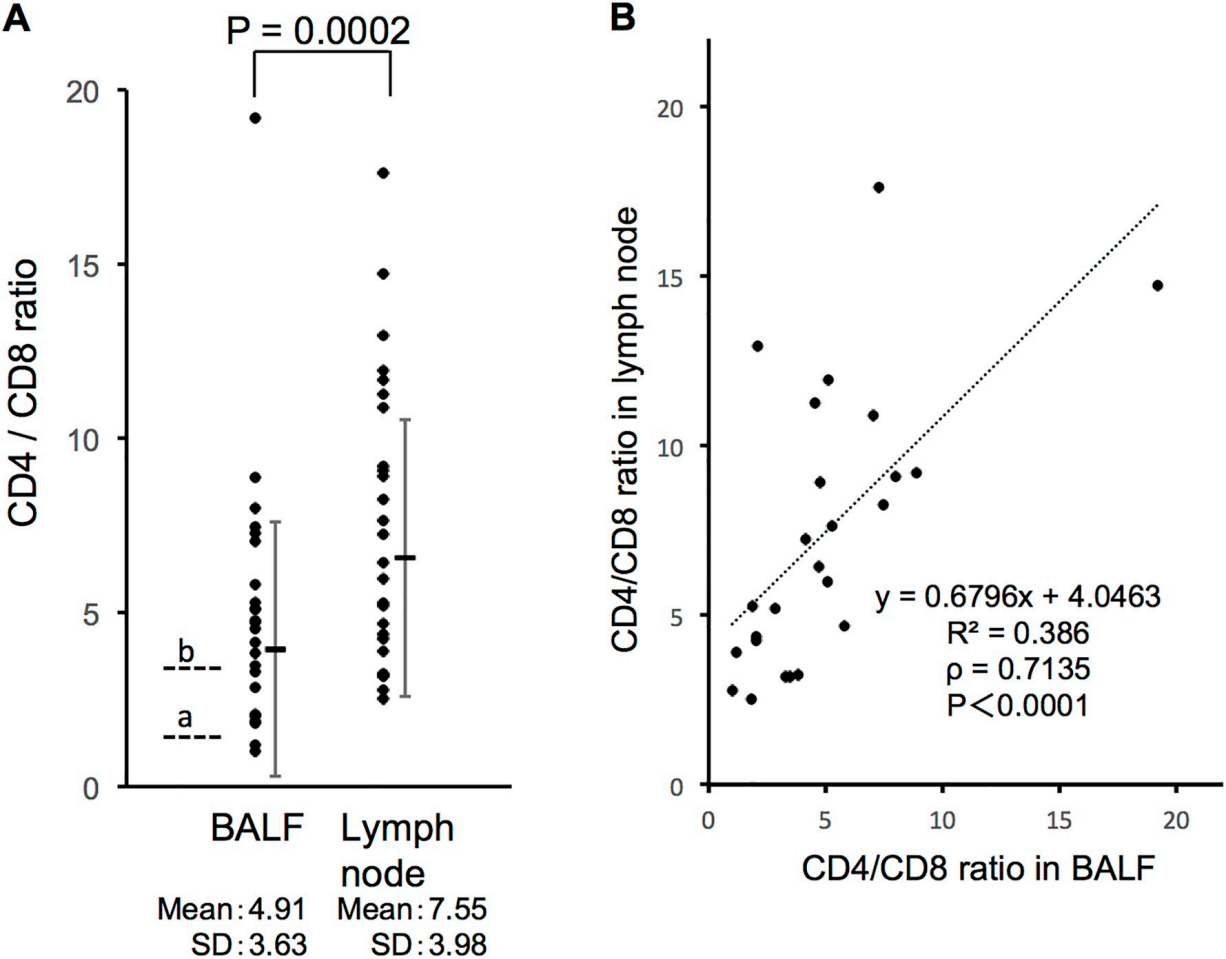
Comparison of the CD4/CD8 ratio in lymph nodes and BAL fluid. (A) The CD4/CD8 ratio was significantly higher in lymph nodes than in BAL fluid (p < 0.0002, Wilcoxon signed-rank test). To evaluate BAL cells, we used the cut-off values (1.34: optimal cut-off value and 3.5: cut-off value with high specificity) reported in [2]: 92.3% and 61.5% of patients fell above the cut-off, respectively. The dashed line (a) indicates the cut-off value of 1.34 and the dashed line (b) indicates the cut-off value of 3.5. (B) The CD4/CD8 ratio in lymph nodes and BAL fluid were significantly co-related to each other (p < 0.0001, Spearman’s rank correlation coefficient).

**Fig 3:**
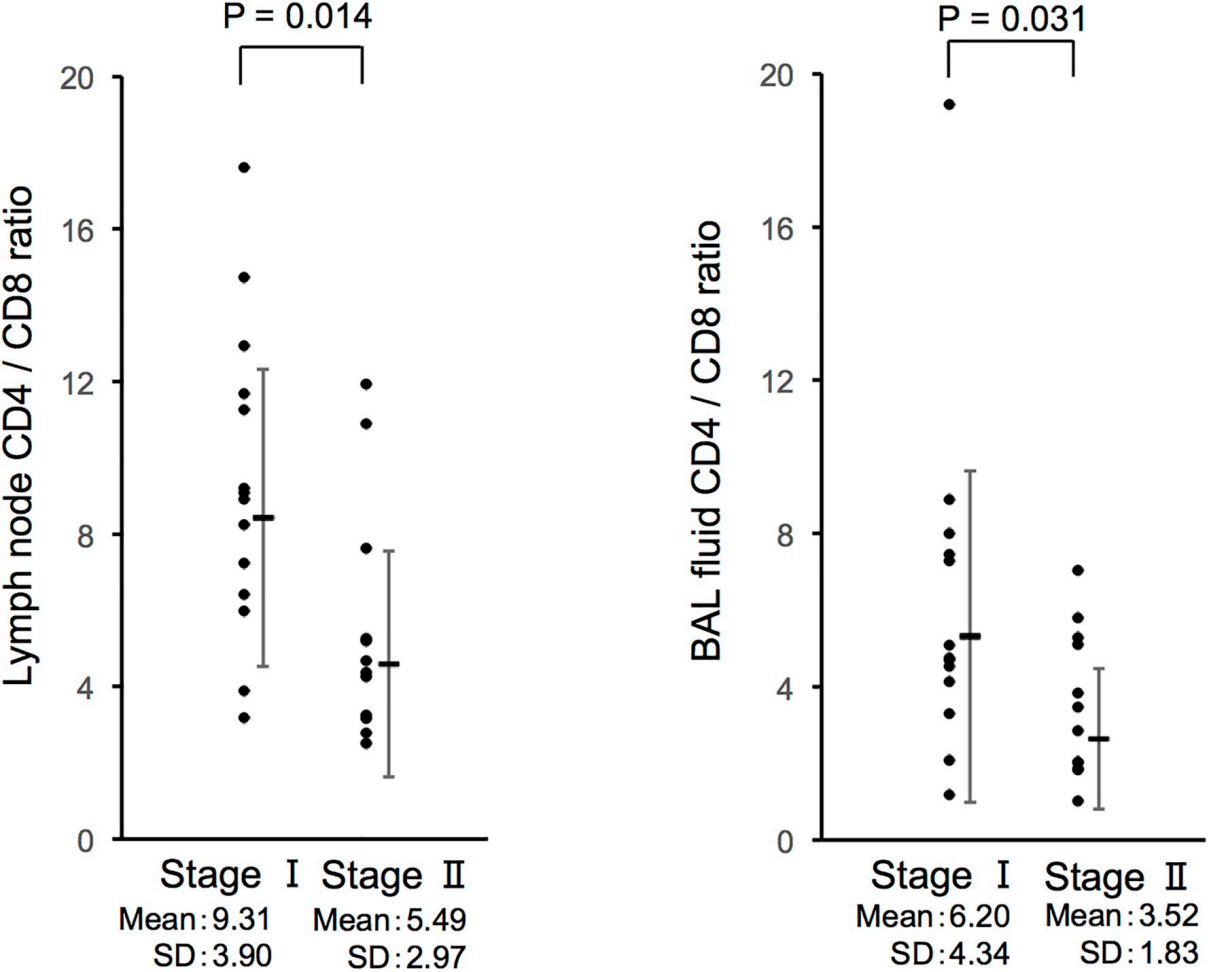
Comparison of the CD4/CD8 ratio by radiographic stage of sarcoidosis in lymph nodes and BAL fluid. The CD4/CD8 ratio was higher in Stage I compared with Stage II. The difference between stages was more significant in lymph nodes than in BAL fluid (p = 0.014, Mann-Whitney *U*-test).

The CD4/CD8 ratio was significantly higher in lymph nodes than in the BAL fluid (p < 0.0002, Wilcoxon signed-rank test). CD4/CD8 ratios in the lymph nodes and BAL fluid were significantly correlated (p < 0.0001, Spearman’s rank correlation coefficient) (Fig 2). Tanriverdi et al. reported that the optimal cut-off value of CD4/CD8 to distinguish from other interstitial lung diseases was 1.34 and that a cut-off value of CD4/CD8 > 3.5 has high specificity (more than 95%) [2]. When we applied both values (1.34 and 3.5) to our BAL CD4/CD8 ratio in sarcoidosis patients, 92.3% and 61.5% of patients fell above the cut-off value, respectively. Interestingly, the CD4/CD8 ratio in lymph node samples was significantly higher in Stage I than in Stage II (p = 0.014, Mann-Whitney *U*-test) (Fig 3). The CD4/CD8 ratio in the BAL fluid of Stage I was also higher than in Stage II but the difference between the two stages was smaller compared to the difference observed in lymph nodes. As shown in Table 2, all sarcoidosis patients (except one with a history of autoimmune pancreatitis) had not received systemic steroid therapy. Thus, the CD4/CD8 ratio difference between Stage I and II in our study was independent of steroid therapy. Furthermore, the CD4/CD8 ratio in patients with or without extrapulmonary lesions was 6.43 ± 2.51 and 7.96 ± 4.33, respectively (mean ± SD). There was no significant difference between the two groups (p = 0.089, Mann-Whitney *U*-test).

### Comparative analysis of the lymphocyte profile of lymphadenopathy between sarcoidosis and lung cancer samples

**Fig 4:**
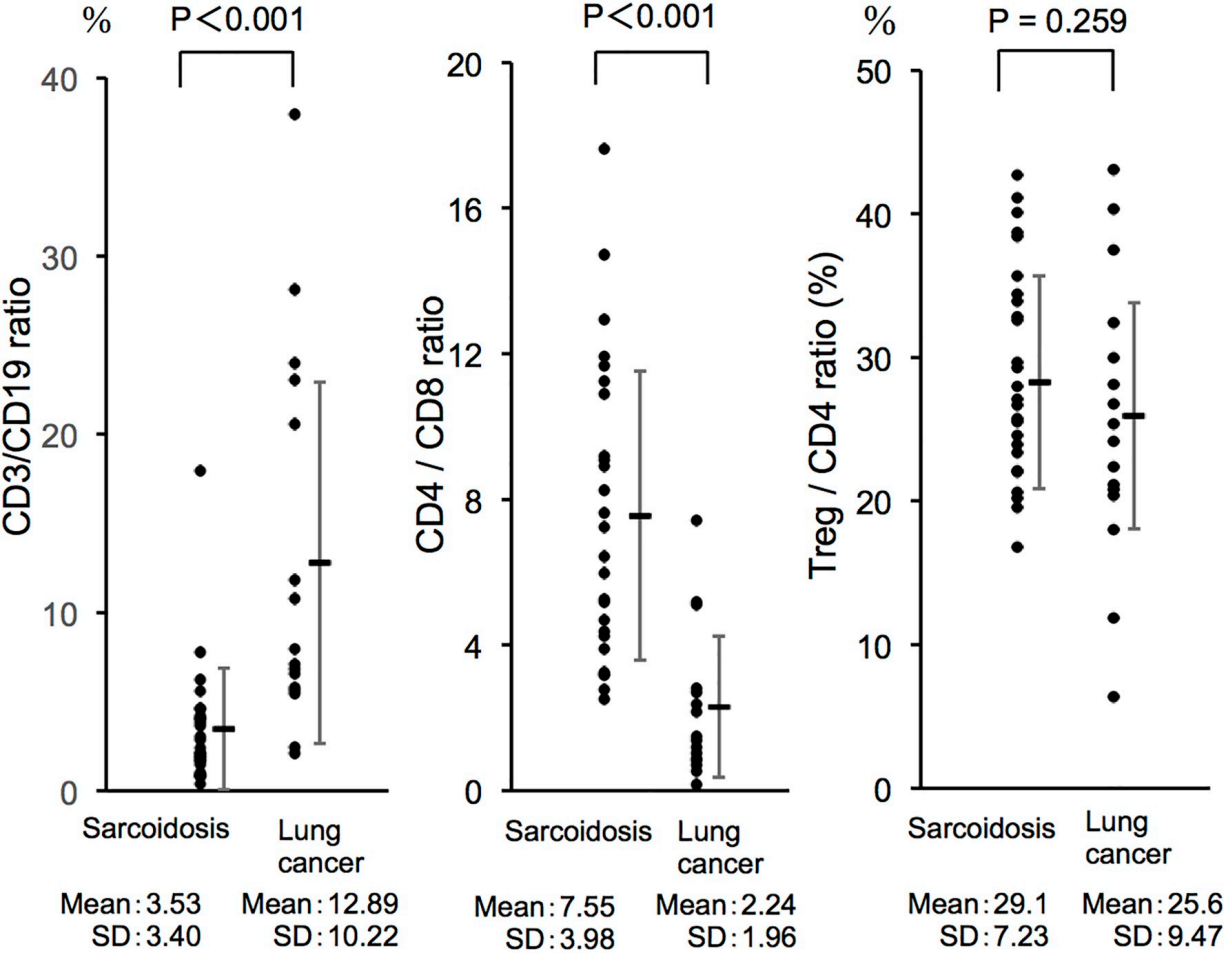
Comparative analyses of the lymphocytes profile in lymphadenopathy between sarcoidosis and lung cancer patients. The CD3/CD19 ratio was significantly higher in lung cancer than sarcoidosis (p < 0.001, Mann-Whitney *U*-test). In contrast, the CD4/CD8 ratio was significantly higher in sarcoidosis than lung cancer (p < 0.001, Mann-Whitney *U*-test). The Treg cell/CD4 cell ratio was not statistically different between sarcoidosis and lung cancer.

Finally, we compared the lymphocyte profile in sarcoidosis lymph nodes with those of lung cancer lymph nodes (five cases with small cell carcinoma, nine with adenocarcinoma, one with squamous cell carcinoma and one with “not otherwise specified” (NOS)). The detailed clinical information of lung cancer patients is shown in Table 3. As shown in Fig 4, the CD3/CD19 (T-lymphocytes /B-lymphocytes) ratio was markedly different between the two diseases. In sarcoidosis, the relative B-lymphocytes proportion was significantly higher than that of lung cancer metastatic lymph nodes. In addition, the CD4/CD8 ratio was significantly higher in sarcoidosis (p < 0.001, Mann-Whitney *U*-test) (Fig 4, Table 4). In contrast, no significant difference was observed in the ratio of Treg /CD4 T-lymphocytes between the two diseases (Fig 4, Table 5). As a larger number of younger patients was included in the sarcoidosis group, we assessed the influence of patient age to the T-lymphocyte profile. We compared the CD3/CD19, CD4/CD8, and Treg/CD4 ratios in lymph nodes from age-matched patients (60–79 years old) from two groups (sarcoidosis and lung cancer). As shown in S2 Fig, the results were the same as those from the analyses of all patients. The CD4/CD8 ratio was still higher in sarcoidosis than lung cancer even when we compared patients whose ages were 60–80 years (sarcoidosis 9.31 ± 4.15, lung cancer 1.70 ± 1.34, mean ± SD) (p < 0.03, Mann-Whitney *U*-test) (S2 Fig). Similarly, we compared the T-lymphocyte profiles in both diseases by gender (S3 and S4 Figs). This revealed that the T-lymphocyte profiles were not influenced by gender differences. Furthermore, there were no significant differences in the CD3/CD19, CD4/CD8, and Treg/CD4 T-lymphocytes ratios between small cell carcinoma and adenocarcinoma, although this data should be treated with caution because of the small sample size (S5 Fig).

**Table 3:** Lung cancer; clinical, radiologic, and pathologic findings.

Case	Age/Sex*	Tissue type**	Stage	Other Organ Metastases§	Primary site	Past medical history
C1	41/M	Ad	cT3N2M1b Stage IV	Costal bone	Lt upper lobe	None
C2	65/F	Ad	cT4N3M1a Stage IV	Bilateral PE Cardiac effusion	Lt hilum	Deep vein thrombosis
C3	83/M	Ad	cT2aN3M0 Stage IIIB	None	Lt lower lobe	Hypertension
C4	69/M	Ad	cT1aN3M1b Stage IV	Costal bone	Rt upper lobe	Prostate cancer Myocardial infarction
C5	77/M	Sm	cT4N3M1b Stage IV ED	Lt PE	Lt hilum	Hypertension
C6	68/F	Sm	cT3N3M1b Stage IV ED	Rt vocal cord	Lt hilum	Stomach cancer Hypertension
C7	89/M	Sm	cT2bN3M1b Stage IV ED	Rt PE Multiple bones	Rt lower lobe	Atrial fibrillation Hypertension
C8	69/M	Ad	cT4N3M1b Stage IV	Lt adrenal gland	Lt upper lobe	Atrial fibrillation Hypertension
C9	81/F	Ad	pT1bN2M0 Post operative recurrence	None	Rt lower lobe	Bronchial asthma Rheumatoid arthritis
C10	77/F	Ad	pT2aN2M0 Post operative recurrence	None	Rt upper lobe	Lt kidney cancer
C11	78/M	Ad	pT3N0M0 Post operative recurrence	None	Rt lower lobe	Bladder cancer Rt parotid tumor
C12	81/M	NOS	cT1aN3M0 Stage IIIB	None	Rt upper lobe	Colon cancer Cerebral infarction
C13	80/M	Sq	cT4N3M0 Stage IIIB	None	Rt upper lobe	Hypertension
C14	44/F	Ad	pT1aN0M0 Post operative recurrence	None	Lt upper lobe	Hyperthyroidism
C15	73/M	Sm	cT3N2M1c Stage IV ED	Celiac lymph nodes Liver	Rt upper lobe	DLBCL Angina pectoris
C16	69/M	Sm	cT2aN3M1c Stage IV ED	Multiple bones Liver	Rt upper lobe	Cerebral infarction Hypertension

Case numbers correspond to those in Tables 4 and 5. All cases of lymph node metastasis were pathologically confirmed by endobronchial ultrasound-guided transbronchial needle aspiration. Cases 9, 10, 11, and 14 underwent biopsy with endobronchial ultrasound-guided transbronchial needle aspiration at postoperative recurrence. Clinical Stage, Other Organ Metastasis, and Chest CT indicate the condition at the time of lung cancer diagnosis. Steroid medication shows the condition at the time of biopsy.

*M, male; F, female

** Sm, small cell carcinoma;

Ad, adenocarcinoma; Sq, squamous cell carcinoma; NOS: not otherwise specified.

^§^ Lt, left;

PE, pleural effusion; Rt, right; DLBCL, diffuse large B-cell lymphoma.

**Table 4:** CD4/CD8 ratio in mediastinal lymph nodes and BAL fluid.

Sarcoidosis			Lung Cancer	
Case*	Mediastinal lymph node	BALF	Case*	Mediastinal lymph node
1	2.78	1.01	C1	2.70
2	3.89	1.19	C2	0.82
3	5.26	1.87	C3	0.17
4	5.98	5.09	C4	0.88
5	7.63	5.28	C5	2.16
6	8.24	7.46	C6	5.18
7	9.07	8.00	C7	2.37
8	6.43	4.71	C8	0.71
9	4.68	5.81	C9	7.42
10	10.89	7.04	C10	1.03
11	4.25	2.03	C11	0.54
12	7.24	4.14	C12	1.20
13	11.26	4.54	C13	1.37
14	17.62	7.28	C14	5.11
15	2.51	1.83	C15	1.48
16	5.19	2.85	C16	2.81
17	14.73	19.19		
18	11.68	NA		
19	11.94	5.11		
20	3.17	3.47		
21	8.91	4.76		
22	3.19	3.30		
23	3.24	3.84		
24	4.37	2.04		
25	9.20	8.88		
26	12.94	2.08		

Numbers correspond to case numbers in Tables 2 and 3. BAL. bronchoalveolar lavage; NA, not available.

**Table 5:** Treg/CD4 ratio in mediastinal lymph nodes.

Sarcoidosis		Lung cancer	
Case*	Treg/CD4 (%)	Case*	Treg/CD4 (%)
1	38.44	C1	28.12
2	35.7	C2	43.08
3	20.63	C3	37.5
4	29.65	C4	40.34
5	33.92	C5	11.85
6	22.01	C6	29.99
7	23.37	C7	26.79
8	19.54	C8	25.37
9	16.81	C9	32.41
10	25.51	C10	20.81
11	40.11	C11	6.38
12	22.1	C12	20.41
13	41.12	C13	18.02
14	26.69	C14	24.19
15	23.94	C15	22.39
16	32.6	C16	21.13
17	25.74		
18	34.41		
19	38.69		
20	27.99		
21	29.29		
22	42.73		
23	20.22		
24	27.09		
25	32.85		
26	24.59		

* Numbers correspond to case numbers in Tables 2 and 3. Treg, regulatory T cell.

## Discussion

With the use of EBUS-TBNA, lymph node biopsy by EBUS-TBNA has become a routine examination for hilar and mediastinal lymphadenopathy in the field of respiratory medicine [13]. Although EBUS-TBNA has some complications, needle biopsy can be performed safely and less invasively compared with surgical procedures such as mediastinoscopy [14]. One of the advantages of EBUS-TBNA is that one can easily obtain fresh samples directly from pathogenic lymph nodes at most stations in the mediastinum [15]. To analyze lymphocyte profiles, it is clearly better to use freshly obtained samples than archived formalin fixed samples.

Many studies have shown that chest radiographic stage is an indicator of sarcoidosis prognosis [16]. Our study showed that the CD4/CD8 ratio in lymph node samples was significantly different by chest radiographic stage, suggesting that the lymphocyte profile is related to the pathology and prognosis of sarcoidosis. In addition, when sarcoidosis was compared with lung cancer, the CD4/CD8 ratio was much higher in sarcoidosis, and the CD3/CD19 ratio was higher in lung cancer indicating a marked difference in the immune system in both diseases. These data suggest samples obtained directly from the lymph nodes are very informative and are expected to help understand the pathological condition of lymphadenopathy.

Regulatory T cells play an important role in many immune-pathologic diseases including various inflammatory diseases and malignancies [17]. Intriguingly, we observed no difference in the percentage of Treg between sarcoidosis and lung cancer. Previous studies have shown sarcoidosis was associated with Treg subset amplification, which had a reduced activity [18, 19]. Other studies proposed Treg cells in sarcoidosis have a regulatory role to control disease activity or establishment anergy [20]. Our data and that of other studies support the idea that Treg cells have an important role in the pathogenesis of sarcoidosis [21]. Recently, more advanced analysis of mediastinal lymph nodes using flow cytometry showed that Th17 cells have an important role in sarcoidosis pathogenesis [22, 23, 24] To elucidate the pathogenic role of the Th1/Th17/Treg balance in our cohort should be the next step of our study [24].

Previous studies of the lymphocyte profile in lymphadenopathy of sarcoidosis showed markedly different results for the CD4/CD8 ratio. Oda et al. reported that the CD4/CD8 ratio in sarcoidosis lymph nodes was lower than that in the BAL fluid using immunohistochemical staining [4]. In addition, Darlington et al. reported that the CD4/CD8 ratio in EUS-FNA samples from sarcoidosis lymph nodes was significantly lower than in BAL fluid samples [25]. They used flow cytometric analyses for their needle aspiration sample, which was the same method used in the current study. In contrast, our study showed the CD4/CD8 ratio was significantly higher in TBNA samples (lymph nodes) than in the BAL fluid. In 84% of sarcoidosis patients in our study, the CD4/CD8 ratio was higher in lymph nodes than the BAL. In another report, the CD4/CD8 ratio in EBUS-TBNA samples was highly variable without any specific tendency [3]. In that study, the CD4/CD8 ratio in lymph nodes was higher than in the BAL fluid in 48% of the studied patients. Although we could not elucidate the reason for these discrepancies, we hypothesize that it might be related to the different methods of analyses (flow cytometric analysis versus immunohistochemical analysis) or different ethnic populations (Caucasian or Japanese populations) [26]. However, even though there is a marked difference in the CD4/CD8 ratio in lymph nodes among these studies, most of them including our study are consistent with the idea that the CD4/CD8 ratio in the BAL fluid and lymph nodes have a strong correlation. This suggests the CD4/CD8 ratio might reflect the pathology of sarcoidosis as a systemic inflammatory and immunologic disorder.

There were some limitations in our study. First, the sample size was relatively small, leading to the possibility of selection bias. Especially, lung cancer metastatic lymph nodes may exhibit various immune-cell profiles according to the histology of lung cancer or patients’ condition. Thus, more patients are required for the detailed analysis of lung cancer metastatic lymph nodes. Second, because we focused only on sarcoidosis and lung cancer metastases, the lymphocyte profile in other benign diseases such as infectious lymphadenitis are unknown. Whether lymphocyte profiles can be used to distinguish sarcoidosis from other benign lymphadenopathies may be an interesting clinical issue. Third, this study was performed at a single academic center in Japan. Although we used the generalized and well-accepted procedures of EBUS-TBNA and flow cytometric analyses, it may be difficult to exclude potential institutional bias. Furthermore, ethnic bias might have a large influence on inflammation during sarcoidosis. Our study suggests Japanese sarcoidosis patients are a relatively homogenous cohort who present with high a CD4/CD8 ratio of T-lymphocytes in the lymph nodes. Multi-centered and global studies including various ethnic groups under the same protocol are warranted for a better understanding of the immunological profile of sarcoidosis.

## Conclusion

Lymphocyte phenotypic analysis of lymphadenopathy was performed using flow cytometry. A high CD4/CD8 ratio was more notable in lymph nodes than in BAL fluid. The lymphocyte profile in sarcoidosis lymph nodes was distinctive from that in lung cancer metastasis. Flow cytometry analysis of lymph nodes might help to clarify the pathophysiology of diseases accompanying lymphadenopathy.

## Supporting information

S1 Fig Representative flowcytometric data of FOXP3. TBNA samples were processed and dispersed into a single cell suspension. Samples were labeled with anti-CD4-FITC + anti-CD25-PE + anti-FoxP3-APC antibodies (Miltenyi Biotec Inc., Bergisch, Gladbach, Germany, diluted 1:10). For FoxP3 labeling, cell fixation and permeabilization treatments were performed. CD4+CD25+ double positive cells were sorted and analyzed for their FOXP3 expression. A rectangular frame indicates positive FOXP3 gating. The percentage of FOXP3 positive cells/CD4+CD25+ cells is shown in the schema.
(TIFF)

S2 Fig Comparative analyses of lymphocyte profiles in lymphadenopathy between sarcoidosis and lung cancer age matched patients (60–79 years). We compared the CD3/CD19, CD4/CD8, and Treg/CD4 ratios in lymph nodes from age-matched patients (60–79 years) from two groups (sarcoidosis and lung cancer). The results were the same as those from the analyses of all patients (Fig 4). The CD4/CD8 ratio was still higher in sarcoidosis than in lung cancer even when we compared patients aged 60–80 years old (sarcoidosis 9.31 ± 4.15, lung cancer 1.70 ± 1.34, mean ± SD) (p < 0.03, Mann-Whitney *U*-test).
(TIFF)

S3 Fig Gender difference in the lymphocyte profile of lymphadenopathy in sarcoidosis. Comparative analyses of T-lymphocyte profiles by gender in sarcoidosis. There was no difference in the CD3/CD19, CD4/CD8 or Treg/CD4 ratios by gender (Mann-Whitney *U*-test).
(TIFF)

S4 Fig Gender difference in the lymphocyte profile of lymphadenopathy in lung cancer. Comparative analyses of T-lymphocyte profiles by gender in lung cancer. There was no difference in the CD3/CD19, CD4/CD8 or Treg/CD4 ratios by gender (Mann-Whitney *U*-test).
(TIFF)

S5 Fig Difference in lymphocyte profile of lymphadenopathy between adenocarcinoma and small cell carcinoma. Comparative analyses of T-lymphocyte profiles by pathological type in lung cancer (adenocarcinoma vs small cell carcinoma). No significant difference was observed by pathological classification (Mann-Whitney *U*-test).
(TIFF)

S1 Protocol A detailed experimental protocol for the analysis of lymphocyte profile in mediastinal lymph nodes (TBNA samples) by flow cytometry. (DOCX)

## References

[1] Hunninghake GW, Crystal RG. Pulmonary sarcoidosis: a disorder mediated by excess helper T-lymphocyte activity at sites of disease activity. N Engl J Med. 1981;305:429–434. 10.1056/NEJM198108203050804 6454846 10.1056/NEJM198108203050804

[2] Tanriverdi H, Uygur F, Ornek T, Erboy F, Altinsoy B, Atalay F, et al Comparison of the diagnostic value of different lymphocyte subpopulations in bronchoalveolar lavage fluid in patients with biopsy proven sarcoidosis. Sarcoidosis Vasc Diffuse Lung Dis. 2016;32:305–312. 26847097

[3] Ruiz SJ, Zhang Y, Mukhopadhyay S. CD4/CD8 Ratio in Mediastinal Lymph Nodes Involved by Sarcoidosis: Analysis of Flow Cytometry Data Obtained by Endobronchial Ultrasound-guided Transbronchial Needle Aspiration. s. 2016;23:288–297. 10.1097/LBR.0000000000000311 27479012 10.1097/LBR.0000000000000311

[4] Oda K, Ishimoto H, Yatera K, Yamada S, Nakao H, Ogoshi T, et al Relationship between the ratios of CD4/CD8 T-lymphocytes in the bronchoalveolar lavage fluid and lymph nodes in patients with sarcoidosis. Respir Investig. 2014;52:179–183. 10.1016/j.resinv.2013.12.003 24853018 10.1016/j.resinv.2013.12.003

[5] Nakajima T, Yasufuku K, Fujiwara T, Yoshino I. Recent advances in endobronchial ultrasound-guided transbronchial needle aspiration. Respir Investig. 2016;54:230–236. 10.1016/j.resinv.2016.02.002 27424821 10.1016/j.resinv.2016.02.002

[6] Fielding D, Kurimoto N. Endobronchial Ultrasound-Guided Transbronchial Needle Aspiration for Diagnosis and Staging of Lung Cancer. Clin Chest Med. 2018;39:111–123. 10.1016/j.ccm.2017.11.012 29433708 10.1016/j.ccm.2017.11.012

[7] Navani N, Nankivell M, Lawrence DR, Lock S, Makker H, Baldwin DR, et al Lung cancer diagnosis and staging with endobronchial ultrasound-guided transbronchial needle aspiration compared with conventional approaches: an open-label, pragmatic, randomised controlled trial. Lancet Respir Med. 2015;3:282–289. 10.1016/S2213-2600(15)00029-6 25660225 10.1016/S2213-2600(15)00029-6 PMC4648022

[8] Sadaf S, Loya A, Akhtar N, Yusuf MA. Role of endoscopic ultrasound-guided-fine needle aspiration biopsy in the diagnosis of lymphoma of the pancreas: A clinicopathological study of nine cases. Cytopathology. 2017;28:536–541. 10.1111/cyt.12442 28737285 10.1111/cyt.12442

[9] Wang J, Chen Q, Wu X, Wang Y, Hou W, Cheng B, Role of endoscopic ultrasound-guided fine-needle aspiration in evaluating mediastinal and intra-abdominal lymphadenopathies of unknown origin. Oncol Lett. 2018;15:6991–6999. 10.3892/ol.2018.8253 29725426 10.3892/ol.2018.8253 PMC5920145

[10] Ribeiro A, Vazquez-Sequeiros E, Wiersema LM, Wang KK, Claim JE, Wiersema MJ. EUS-guided fine-needle aspiration combined with flow cytometry and immunocytochemistry in the diagnosis of lymphoma. Gastrointest Endosc. 2001;53:485–491. 10.1067/mge.2001.112841 11275890 10.1067/mge.2001.112841

[11] Rusch VW, Asamura H, Watanabe H, Giroux DJ, Rami-Porta R, Goldstraw P, The IASLC lung cancer staging project -a proposal for a new international lymph node map in the forthcoming seventh edition of the TNM classification for lung cancer. J Thorac Oncol. 2009; 4: 568–577. 10.1097/JTO.0b013e3181a0d82e 19357537 10.1097/JTO.0b013e3181a0d82e

[12] Meyer KC, Raghu G, Baughman RP, Brown KK, Costabel U, du Bois RM, et al An official American Thoracic Society clinical practice guideline: the clinical utility of bronchoalveolar lavage cellular analysis in interstitial lung disease. Am J Respir Crit Care Med. 2012;185:1004–1014. 10.1164/rccm.201202-0320ST 22550210 10.1164/rccm.201202-0320ST

[13] Trisolini R, Lazzari Agli L, Tinelli C, De Silvestri A, Scotti V, Patelli M, Endobronchial ultrasound-guided transbronchial needle aspiration for diagnosis of sarcoidosis in clinically unselected study populations. Respirology. 2015;20:226–234. 10.1111/resp.12449 25477156 10.1111/resp.12449

[14] Agarwal R, Srinivasan A, Aggarwal AN, Gupta D, Efficacy and safety of convex probe EBUS-TBNA in sarcoidosis: a systematic review and meta-analysis. Respir Med. 2012;106:883–892. 10.1016/j.rmed.2012.02.014 22417738 10.1016/j.rmed.2012.02.014

[15] Imai N, Imaizumi K, Ando M, Shimokata T, Ogawa T, Ito S, et al Echoic features of lymph nodes with sarcoidosis determined by endobronchial ultrasound. Intern Med. 2013;52:1473–1478. 23812194 10.2169/internalmedicine.52.9082

[16] Nunes H, Uzunhan Y, Gille T, Lamberto C, Valeyre D, Brillet PY. Imaging of sarcoidosis of the airways and lung parenchyma and correlation with lung function. Eur Respir J. 2012;40:750–765. 10.1183/09031936.00025212 22790910 10.1183/09031936.00025212

[17] Moldaver DM, Larche M, Rudulier CD. An Update on Lymphocyte Subtypes in Asthma and Airway Disease. Chest. 2017;151:1122–1130. 10.1016/j.chest.2016.10.038 27818326 10.1016/j.chest.2016.10.038 PMC6026230

[18] Miyara M, Amoura Z, Parizot C, Badoual C, Dorgham K, Trad S, et al The immune paradox of sarcoidosis and regulatory T cells. J Exp Med. 2006;203:359–370. 10.1084/jem.20050648 16432251 10.1084/jem.20050648 PMC2118208

[19] Moller DR, Rybicki BA, Hamzeh NY, Montgomery CG, Chen ES, Drake W, et al Genetic, Immunologic, and Environmental Basis of Sarcoidosis. Ann Am Thorac Soc. 2017;14(Supplement 6):S429–S436. 10.1513/AnnalsATS.201707-565OT 29073364 10.1513/AnnalsATS.201707-565OT PMC5822412

[20] Sakthivel P, Grunewald J, Eklund A, Bruder D, Wahlstrom J. Pulmonary sarcoidosis is associated with high-level inducible co-stimulator (ICOS) expression on lung regulatory T cells—possible implications for the ICOS/ICOS-ligand axis in disease course and resolution. Clin Exp Immunol. 2016;183:294–306. 10.1111/cei.12715 26415669 10.1111/cei.12715 PMC4711163

[21] Broos CE, Hendriks RW, Kool M. T-cell immunology in sarcoidosis: Disruption of a delicate balance between helper and regulatory T-cells. Curr Opin Pulm Med. 2016;22:476–483. 10.1097/MCP.0000000000000303 27379969 10.1097/MCP.0000000000000303

[22] Ramstein J, Broos CE, Simpson LJ, Ansel KM, Sun SA, Ho ME, et al Interferon-gamma-producing Th17.1 cells are increased in sarcoidosis and more prevalent than Th1 cells. Am J Respir Crit Care Med. 2015; 193: 1281**–**1291. 10.1164/rccm.201507-1499OC PMC4910899 26649486

[23] Broos CE., Koth LL., van Nimwegen M, In ‘t Veen JCCM, Paulissen SMJ, van Hamburg JP, et al Increased T-helper 17.1 cells in sarcoidosis mediastinal lymph nodes. Eur Respir J. 2018;51 10.1183/13993003.01124-2017 29449421

[24] Mortaz E, Rezayat F, Amani D, Kiani A, Garssen J, Adcock IM, et al The Roles of T Helper 1, T Helper 17 and Regulatory T Cells in the Pathogenesis of Sarcoidosis. Iran J Allergy Asthma Immunol. 2016;15:334–339. 27921415

[25] Darlington P, Haugom-Olsen H, von Sivers K, Wahlström J, Runold M, Svjatoha V, et al T-cell phenotypes in bronchoalveolar lavage fluid, blood and lymph nodes in pulmonary sarcoidosis—indication for an airborne antigen as the triggering factor in sarcoidosis. J Intern Med. 2012;272:465–471. 10.1111/j.1365-2796.2012.02543.x 22469005 10.1111/j.1365-2796.2012.02543.x

[26] Westney GE, Judson MA. Racial and ethnic disparities in sarcoidosis: from genetics to socioeconomics. Clin Chest Med. 2006;27:453–462. 10.1016/j.ccm.2006.04.002 16880055 10.1016/j.ccm.2006.04.002

