# Supplementary figures and images for: Flow cytometric analysis of lymphocyte profiles in mediastinal lymphadenopathy of sarcoidosis
Source: PLoS One. 2018 Nov 19;13(11):e0206972. doi: 10.1371/journal.pone.0206972 (PMC6242308; doi:10.1371/journal.pone.0206972)

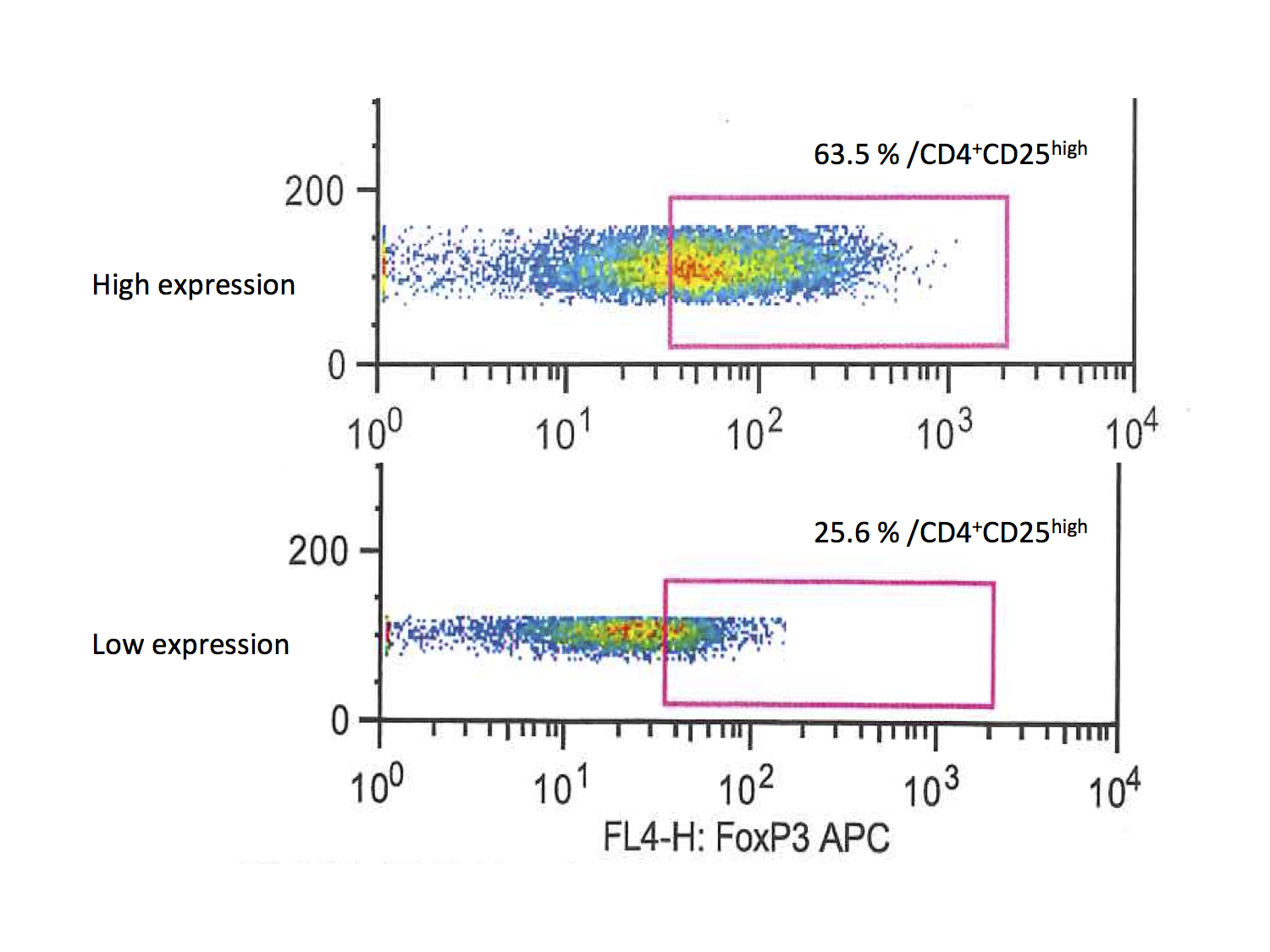

Supplement: S1 Fig — TBNA samples were processed and dispersed into a single cell suspension. Samples were labeled with anti-CD4-FITC + anti-CD25-PE + anti-FoxP3-APC antibodies (Miltenyi Biotec Inc., Bergisch, Gladbach, Germany, diluted 1:10). For FoxP3 labeling, cell fixation and permeabilization treatments were performed. CD4+CD25+ double positive cells were sorted and analyzed for their FOXP3 expression. A rectangular frame indicates positive FOXP3 gating. The percentage of FOXP3 positive cells/CD4+CD25+ cells is shown in the schema. (TIFF) [file pone.0206972.s001.tiff]

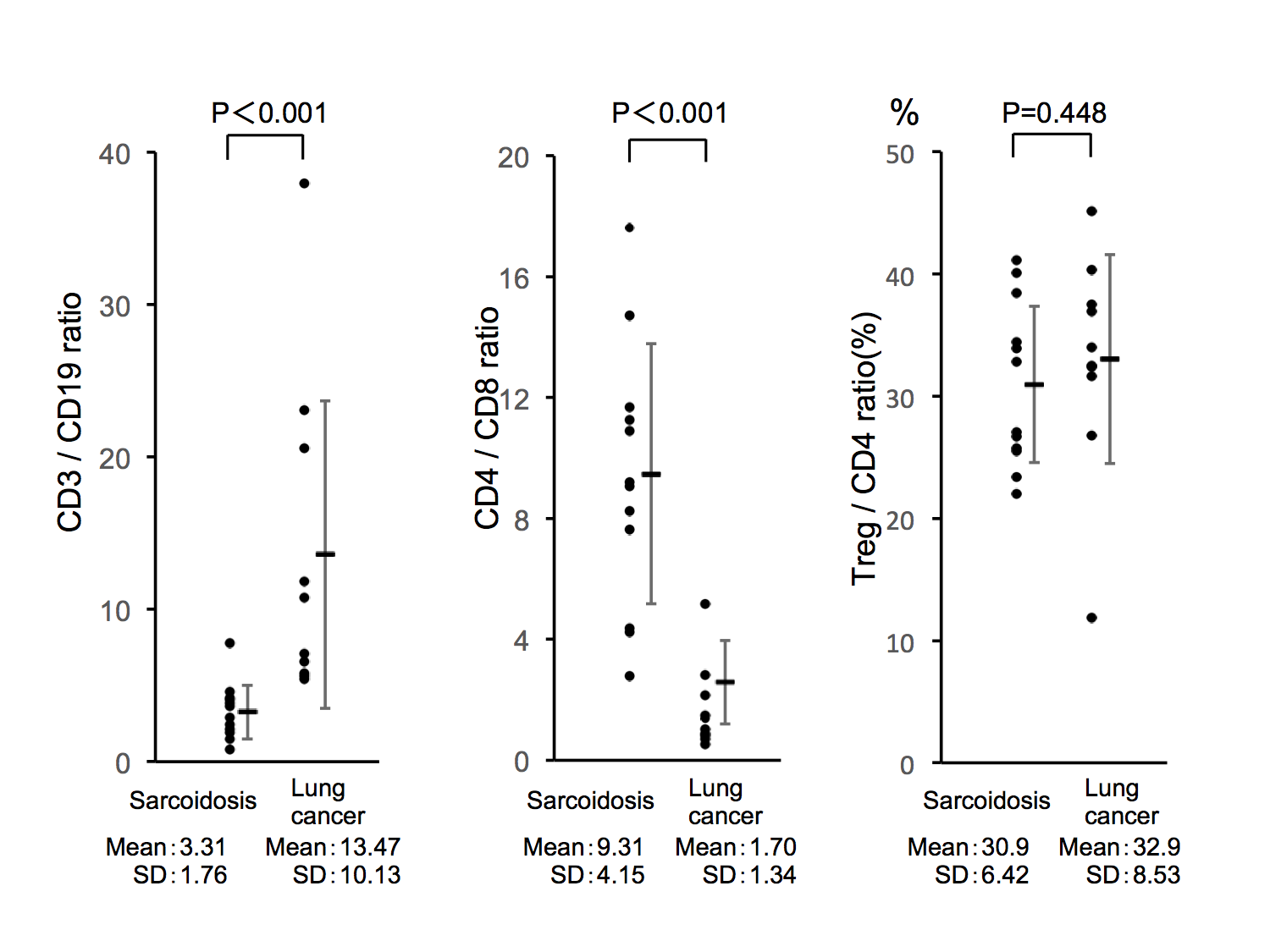

Supplement: S2 Fig — We compared the CD3/CD19, CD4/CD8, and Treg/CD4 ratios in lymph nodes from age-matched patients (60–79 years) from two groups (sarcoidosis and lung cancer). The results were the same as those from the analyses of all patients (Fig 4). The CD4/CD8 ratio was still higher in sarcoidosis than in lung cancer even when we compared patients aged 60–80 years old (sarcoidosis 9.31 ± 4.15, lung cancer 1.70 ± 1.34, mean ± SD) (p < 0.03, Mann-Whitney U-test). (TIFF) [file pone.0206972.s002.tiff]

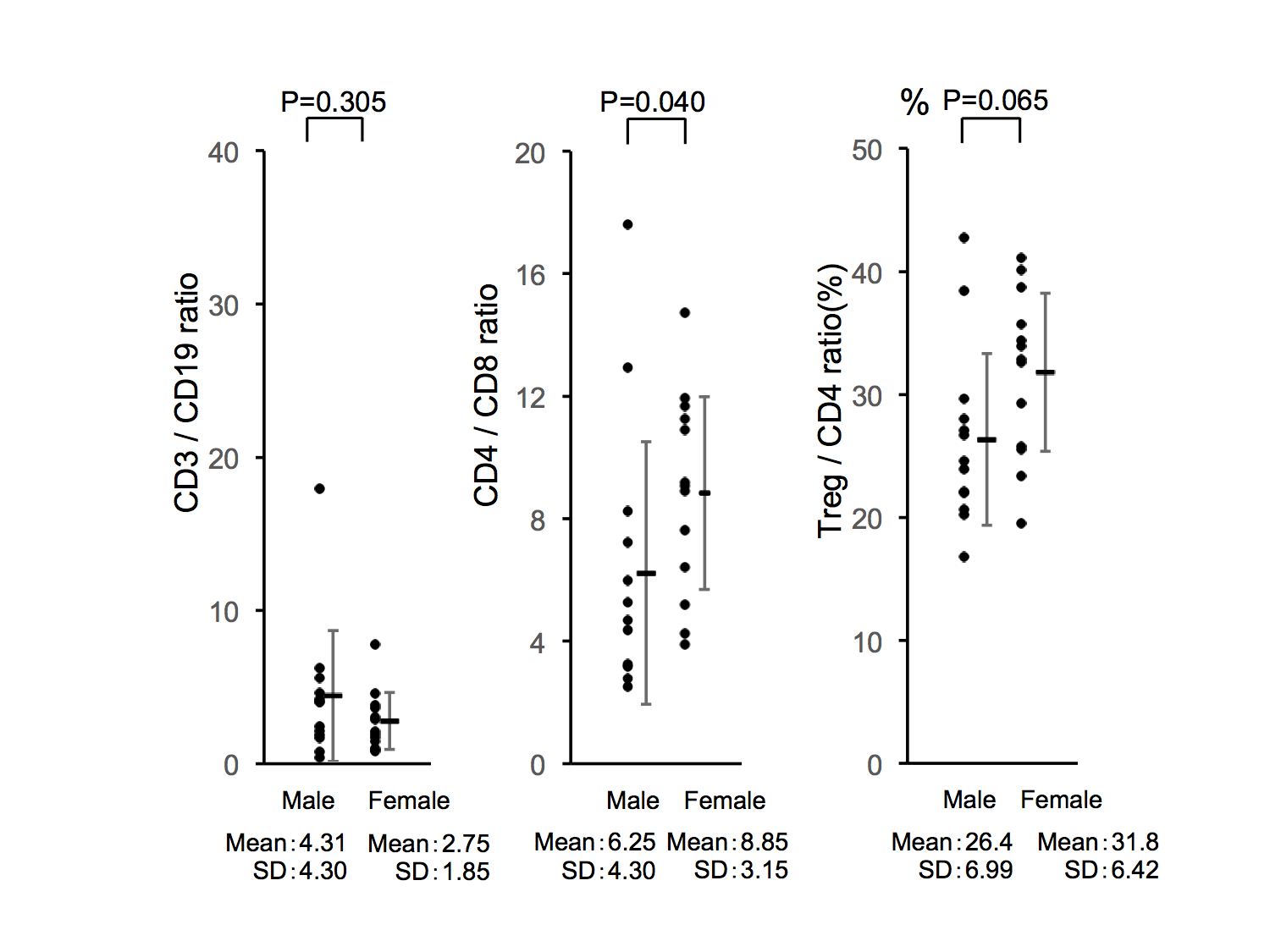

Supplement: S3 Fig — Comparative analyses of T-lymphocyte profiles by gender in sarcoidosis. There was no difference in the CD3/CD19, CD4/CD8 or Treg/CD4 ratios by gender (Mann-Whitney U-test). (TIFF) [file pone.0206972.s003.tiff]

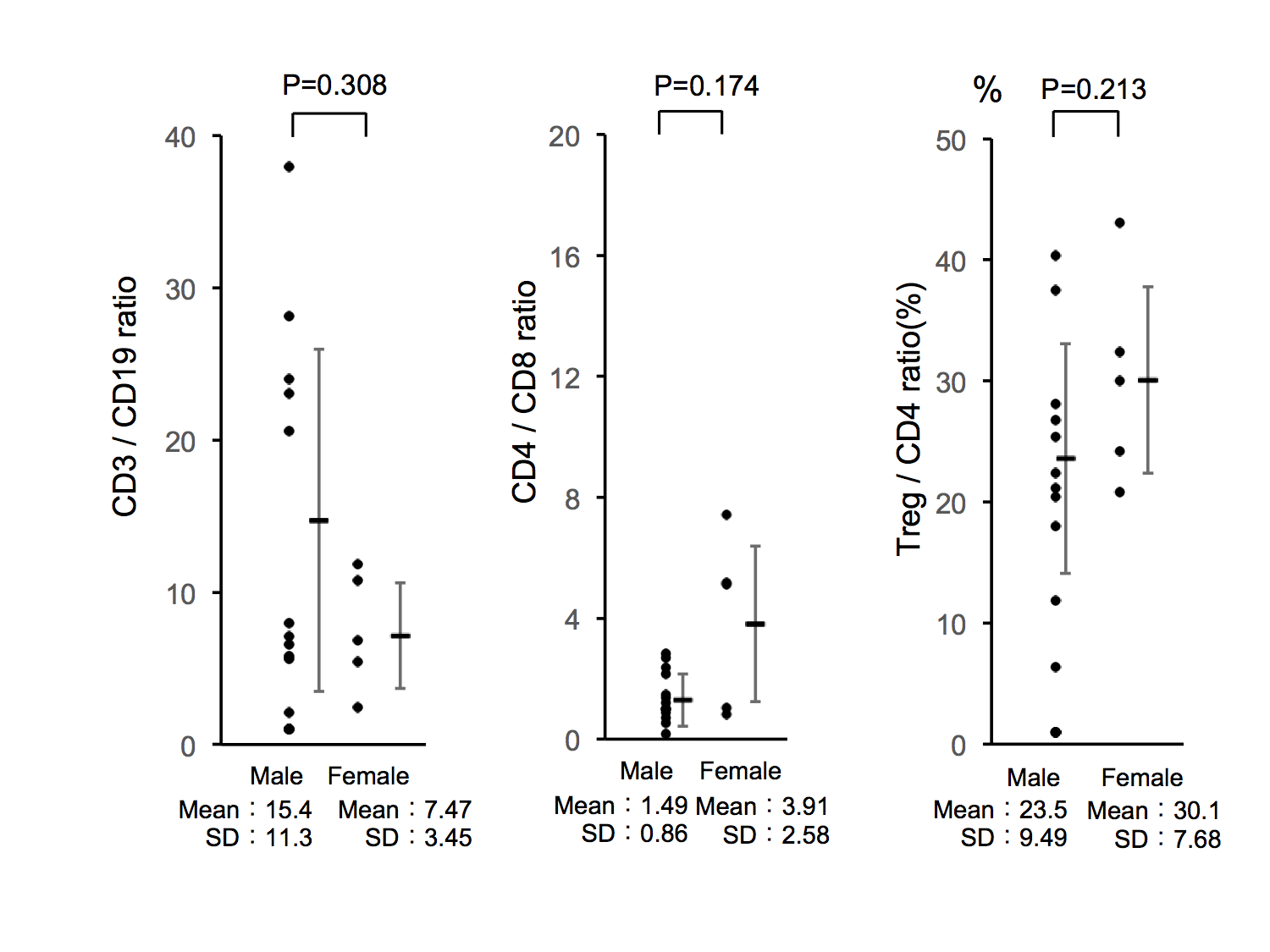

Supplement: S4 Fig — Comparative analyses of T-lymphocyte profiles by gender in lung cancer. There was no difference in the CD3/CD19, CD4/CD8 or Treg/CD4 ratios by gender (Mann-Whitney U-test). (TIFF) [file pone.0206972.s004.tiff]

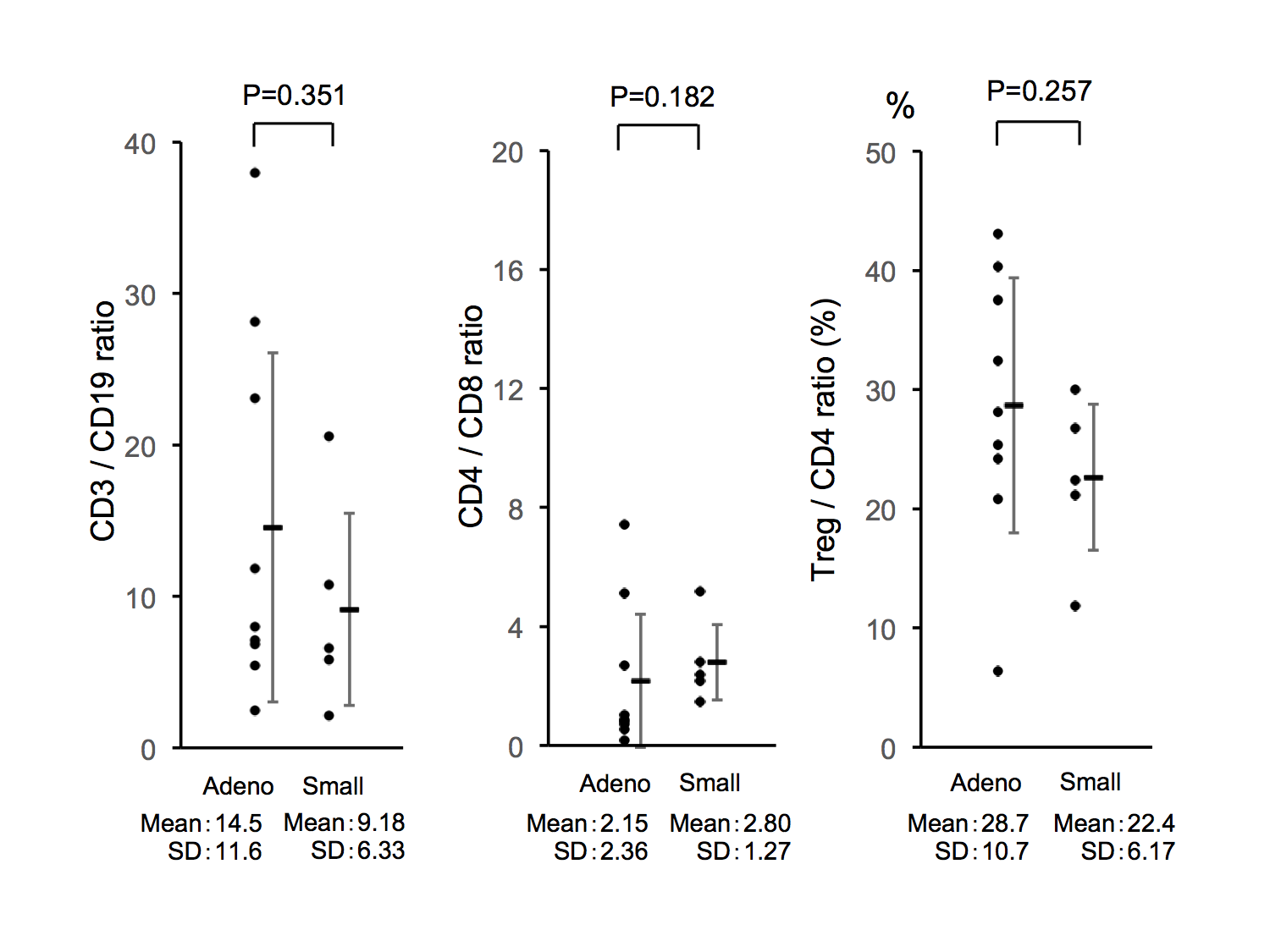

Supplement: S5 Fig — Comparative analyses of T-lymphocyte profiles by pathological type in lung cancer (adenocarcinoma vs small cell carcinoma). No significant difference was observed by pathological classification (Mann-Whitney U-test). (TIFF) [file pone.0206972.s005.tiff]
