# Supplementary material for: Flow cytometric analysis of lymphocyte profiles in mediastinal lymphadenopathy of sarcoidosis
Source: PLoS One. 2018 Nov 19;13(11):e0206972. doi: 10.1371/journal.pone.0206972 (PMC6242308; doi:10.1371/journal.pone.0206972)
Supplement: S1 Protocol — (DOCX) [file pone.0206972.s006.docx]

1) First preparation

・Sample is collected in 5% FBS in 1 ml DMEM high glucose.

・Transfer the sample to a 15 ml centrifuge tube and add PBS containing 10% wash and recovery solution (CELLOTION, Nippon Zenyaku Kogyo Co., Ltd., Fukushima, Japan) to a 10 ml volume.

・For washing, mix by inversion, centrifuge at 150 xg for 5 minutes, discard the supernatant. ①

・Remove sample from the 15 ml centrifuge tube, cut it on a petri dish, return it to the 15 ml centrifuge tube, add PBS and mix.

・Wash again as in ①.

・For hemolysis, add erythrocyte lysing reagent without fixative (EasyLyse Agilent Technologies, Inc., CA, USA), mix by overturning and allow it to react for 15 minutes at room temperature.

・Add PBS and wash as in ①.

・Add PBS to the sample and filtrate to a 50 μl centrifuge tube.

・Centrifuge at 1200 rpm for 5 minutes, discard the supernatant leaving 1 ml.

・Dispense 6 μl, stain with trypan blue and count the number of cells.

・Dispense 50 – 100 μl into each of 8 microtubes for 2 Negative Controls, CD3/CD4/CD8, CD3/CD19, CD4, CD25, FoxP3, and Treg staining.

・Centrifuge at 1200 rpm for 5 minutes and discard the supernatant. Add 90 μl of 0.5% BSA in PSA to each microtube.

2) Measurement of CD4/CD8 ratio and CD3/CD19 ratio

・10 μl of anti-CD3 + anti-CD4 + anti-CD8 antibodies (CD4-FITC/CD8-PE/CD-3-PC5; Beckman Coulter, Inc., CA, diluted 1: 10) was added to the microtube for CD3/CD4/CD8, and anti-CD3 + anti-CD19 antibodies (CD3-FITC/CD-19-PE; Beckman Coulter, Inc., diluted 1: 10) was added to the CD3/CD19 microtube.

・React the three microtubes with one NC at room temperature for 15 minutes.

・Add 1 ml of PBS, centrifuge at 1200 rpm for 5 minutes, transfer to a FC tube and perform flow cytometry.

3) Measurement of regulatory T cells (Treg).

・Treg were measured using a Treg Detection Kit (CD4/CD25/FoxP3 APC), human (Miltenyi Biotec Inc., Bergisch, Gladbach, Germany).

・Add 5 μl of anti-CD4 (VIT 4)-FITC antibodies (diluted 1:10) contained in the kit to the CD4 microtube and 10 μl to the Treg microtube.

・Add 5 μl of anti-CD25 (4E3)-PE antibodies (diluted 1: 10) contained in the kit to the CD25 microtube and 10 μl to the Treg microtube.

・Three microtubes to which antibody has been added are reacted at 4°C for 10 minutes, then 1 ml of 0.5% BSA in PBS is added, followed by centrifugation at 1200 rpm for 5 minutes, and the supernatant is removed.

・Add 500 μl of Fix Buffer contained in the kit to each of 5 microtubes containing the Negative Control and FoxP3 microtubes for fixation and drilling of cells, and react for 30 minutes at 4°C in the dark.

・Centrifuge at 1200 rpm for 5 minutes, remove the supernatant, add 1 ml Permeabilization Buffer contained in the kit, centrifuge again at 1200 rpm for 5 minutes and remove the supernatant.

・Add 80 μl of Permeabilization Buffer contained in the kit to each microtube, then add 20 μl of Blocking Buffer to the Treg microtube and 10 μl to the other microtubes and react at 4°C for 5 minutes.

・Add 5 μl anti-FoxP3 (3G 3)-APC antibodies (diluted 1: 10) contained in the kit to the FoxP3 microtube and 10 μl to the Treg microtube, and react at 4°C for 5 minutes.

・Add 1 ml Permeabilization Buffer to each microtube, centrifuge at 1,500 rpm for 5 minutes, transfer to a FC tube and perform flow cytometry.
